# Enhanced Contextual Fear Memory and Elevated Astroglial Glutamate Synthase Activity in Hippocampal CA1 BChE shRNA Knockdown Mice

**DOI:** 10.3389/fpsyt.2020.564843

**Published:** 2020-09-11

**Authors:** Si Chen, Zhengdong Lin, Kai-Leng Tan, Risheng Chen, Wenfang Su, Haishan Zhao, Qiwen Tan, Wen Tan

**Affiliations:** ^1^ Department of Human Anatomy and Histology & Embryology, Zunyi Medical University, Zhuhai Campus, Zhuhai, China; ^2^ Institute of Biomedical and Pharmaceutical Sciences, Guangdong University of Technology, Guangzhou, China; ^3^ Jeffrey Cheah School of Medicine and Health Sciences, Monash University Malaysia Campus, Bandar Sunway, Malaysia

**Keywords:** butyrylcholinesterase, acetylcholine, hippocampus CA1, posttraumatic stress disorder, contextual fear memory, astrocyte, glutamine synthetase

## Abstract

Butyrylcholinesterase (BChE) efficiently hydrolyzes acetylcholine (ACh) at high concentrations when acetylcholinesterase (AChE) is substrate-inhibited. Recent studies have shown that BChE also has a function that is independent of ACh, but it has not been fully explored. Low BChE expression is accompanied with higher stress-induced aggression and ghrelin levels in stress models, and BChE knockout mice exhibit cognitive and memory impairments. However, the role of BChE in posttraumatic stress disorder (PTSD) remains unclear. In the present study, we investigated the role of BChE in contextual fear memory and its regulatory effect on the expression of factors related to the glutamate (Glu)-glutamine (Gln) cycle *via* knockdown studies. We used AAVs and lentiviruses to knockdown BChE expression in the mouse hippocampal CA1 region and C8D1A astrocytes. Our behavioral data from those mice injected with AAV-shBChE in the hippocampal CA1 region showed strengthened fear memory and increased dendritic spine density. Elevated Glu levels and glutamine synthetase (GS) enzyme activity were detected in contextual fear conditioned-BChE knockdown animals and astrocytes. We observed that an AAV-shBChE induced lowering of BChE expression in the hippocampus CA1 region enhanced contextual fear memory expression and promoted the astrocytic Glu-Gln cycle but did not elevate ACh-hydrolyzing activity. This study provides new insight into the regulatory role of BChE in cognition and suggests potential target for stress-related psychiatric disorder such as PTSD where patients experience fear after exposure to severe life-threatening traumatic events.

## Introduction

High concentration of butyrylcholinesterase (BChE) can efficiently hydrolyzes acetylcholine (ACh) in the mechanism of acquisition and/or expression of contextual freezing responses when acetylcholinesterase (AChE) is substrate-inhibited (1–3). BChE has been largely implicated in cognitive function, as demonstrated in human serology studies (1, 4). Elevated BChE expression has been reported to reduce stress-induced aggression (5–7). Not only has the cholinergic system been shown to be involved in the pathogenesis of posttraumatic stress disorder (PTSD) in clinical studies, but evidence for the involvement of frontal cortex and hippocampus cholinergic pathways in mediating aversive fear memory has been demonstrated in a repeated trauma model in rats (8–10). Nevertheless, the role of BChE in PTSD has been seldom reported. PTSD is a common stress-related psychiatric disorder that may occur in individuals after exposure to severe life-threatening traumatic events (11, 12), such as earthquakes, public health emergencies, and terrible experiences. Additionally, clinical studies have revealed that it is associated with an increased risk for premature mortality, in part due to an increased risk for prevalent diseases such as cardiovascular disease (CVD) and type 2 diabetes mellitus (T2DM) (13, 14). There is a lack of effective pharmacotherapeutic interventions for PTSD (15); thus, it is important to understand the pathological mechanism of PTSD. Contextual fear conditioning (CFC) is a paradigm that mimics the symptoms of PTSD. The hippocampus is involved in contextual fear memory formation and has been highlighted to play a key role in the etiology of PTSD (11, 12). This brain region is involved in the rapid acquisition of CFC and the retrieval of contextual memory after a long time (i.e., 24 h) as well as the consolidation process, which is important for long-term contextual fear memory (12, 16, 17).

The glutamatergic system has also been known to play a major role in the pathogenesis of PTSD (18–20). Like ACh, glutamate (Glu) is a primary excitatory neurotransmitter in the nervous system (21, 22). A total of 70% of synaptic Glu in the brain is produced by the Glu-glutamine (Gln) cycle (23). The Glu-Gln cycle has been widely verified to be involved in memory formation and synaptic transmission. The cycle is dependent on bidirectional communication between neurons and astrocytes, which is conceptualized as the tripartite synapse (22–26). When Glu is released from presynaptic neurons, it is converted to Gln in surrounding astrocytes before being transported back to the presynaptic neuron for reconversion into Glu (18, 27). This cycle is the direct regulator of neural signal transmission, which relies on astrocytes (21, 22). Increasing evidence has validated that BChE has a direct relationship with nonexcitable cells, such as astrocytes. The functions of this relationship are independent of the ACh-hydrolyzing activity (28) and nonenzymatic functions (29) of BChE. Some clinical studies have even suggested that BChE in the brain might be a biomarker for glial fibrillary acidic protein (GFAP) (29). These results indicate that the Glu-Gln cycle, which is dependent on astrocytes, is potentially regulated by BChE.

To investigate the role of BChE expression in contextual fear memory expression, we knocked down BChE expression in the hippocampal CA1 region of mice and subjected the mice to CFC and also extended the BChE knockdown study to an astrocyte cell line. Here, we report that strengthened contextual fear memory was found in the *in vivo* experiment and that increased dendritic spine density, Glu levels and GS enzyme activity without changes in ACh levels were found in both the *in vivo* and *in vitro* experiments. This explicitly shows that BChE is involved in astrocyte-dependent Glu-Gln cycle regulation and is capable of affecting contextual fear memory.

## Methods

### Mice

Six- to seven-week-old C57BL/6 male mice were obtained from Sun Yat-Sen University. All mice were kept on a 12:12-h light:dark cycle and given a standard pellet diet and autoclaved water *ad libitum*. The mice were randomly assigned to experimental groups after a 7-day acclimatization period. All of the animal experiments were performed in accordance with the National Institutes of Health Guide for the Care and Use of Laboratory Animals, and the protocols were approved by the Animal Care and Use Committee of Sun Yat-Sen University (ACUC Approval number: SCXK GUANGDONG 2011-0029).

### Cell Culture

A total of 6 × 10^5^ mouse cloned astrocyte C8D1A cells (ATCC, USA) were seeded into a Corning^®^ 25 cm² cell culture flask. The cells were maintained in high glucose Dulbecco’s modified Eagle’s medium (DMEM) (Gibco, USA) containing 10% fetal bovine serum (Thermo Scientific, USA) and 1% penicillin-streptomycin solution (Thermo Scientific, USA) at 37°C in a humidified atmosphere of 5% CO_2_.

### AAV2/9-shBChE Generation

AAV2/9-shBChE was produced by transfecting 293T cells with three plasmids: an AAV vector expressing BChE shRNA and eGFP or EGFP alone, an AAV helper plasmid (pAAV Helper) and an AAV Rep/Cap expression plasmid. AAV2/9- eGFP served as the control in this experiment. Seventy-two hours after transfection, the cells were collected and lysed using a freeze-thaw procedure. The viral particles were purified by the iodixanol step-gradient ultracentrifugation method. Iodixanol was diluted, and the AAV was concentrated using a 100-kDa molecular mass cutoff ultrafiltration device. The genomic titer was 2.5 × 10^12^ to 3.5 × 10^12^ infectious units per ml, as determined by quantitative PCR. To construct shRNAs, oligonucleotides containing 21-base sense and antisense sequences were connected with a hairpin loop followed by a poly (T) termination signal. The sequence targeting BChE (GenBank accession: NM_009738) that was used in the experiments was 5’- GCTCAGATCTTAGTGGGAGTT-3’. The sequence of the control shRNA was TTCTCCGAACGTGTCACGT. These shRNAs were ligated into an AAV2/9 vector expressing EGFP.

### Stereotactic Virus Injection

Briefly, mice were anesthetized by intraperitoneal injection of a mixture of ketamine (7.0 mg/ml) and xylazine (0.44 mg/ml) dissolved in 0.9% saline. The head of each mouse was placed in a stereotactic apparatus (RWD, China). All the mice undergo the CFC and open-field test were bilaterally microinjected. The mice were bilaterally microinjected at the following coordinates to fill the entire dorsal CA1 region: 2.0 mm anteroposterior (AP) from bregma, ± 1.5 mm mediolateral (ML), and 1.70 mm dorsoventral (DV). To evaluate the viral efficacy *in vivo*, the mice were unilaterally microinjected. For unilateral microinjection, the following coordinates were used to fill one side of the dorsal CA1 region: 2.0 mm anteroposterior (AP) from bregma, + 1.5 mm mediolateral (ML), and 1.70 mm dorsoventral (DV). For *in vivo* viral injections, the viral vectors were targeted to the bilateral hippocampal CA1 region. The needle was lowered into the hippocampal CA1 region, and 0.5 µl of virus was delivered over 20 min. The injection needle was withdrawn 5 min after the second infusion. The mice were used 3 weeks after AAV injection. The injection sites were examined at the end of all the behavior tests, and only data from animals in which the injection placement was correct were included.

### CFC

CFC was performed according to a method described previously (12). The conditioning chamber (Shanghai Xinruan Information Technology, China) was constructed of white plastic (20 ×23 ×20 cm) and had a clear lid. The floor of the chamber consisted of 10 parallel stainless-steel grid bars. The grid was connected to a scrambled shocker to deliver foot shocks. On Day 1, the mice were pre-exposed to the chamber for 5 min. Five minutes later, the mice were exposed to the conditioning chamber for 2 min (habituation phase) and then administered three foot shocks (2-s duration, 0.7 mA, 60-s intershock interval). They remained in the chamber for an additional 1 min. Contextual fear memory was evaluated during the test session 24 h (Day 2) after conditioning by placing the mice in the training environment for 5 min. Memory was assessed and is expressed as the percentage of time that the mice exhibited freezing behavior. An animal was considered to be freezing when it crouched without exhibiting movement of its body or head except movement associated with breathing (30). Such behavior is commonly used as an index of fear in mice. Behavioral data were recorded with digital video cameras, and freezing was quantified from the digitized video images using Shanghai Xinruan software. The chamber was cleaned with 75% alcohol after each session to remove any odorant cues.

### Open-Field Test

Mice were placed in a 50 × 50 × 50 cm Plexiglas box and were allowed to move freely for 10 min (Shanghai Xinruan Information Technology, CN). The box was cleaned with 75% alcohol after each session to remove any odorant cues. An overhead camera placed above the box recorded each session. Locomotor parameters were analyzed with TopScan LITE software (Shanghai Xinruan Information Technology, CN).

### Immunohistochemistry

Three weeks postinjection, the mice were transcardially perfused with cold PBS followed by 4% paraformaldehyde (PFA) in PBS. The brains were extracted, postfixed overnight in 4% PFA at 4°C, and then dehydrated in gradient sucrose solution (in PBS). The brains were sectioned at a thickness of 25 µM using a sliding freezing microtome (VT 1000S, Leica, Germany). The sections were treated with 0.3% H_2_O_2_ in PBS at room temperature for 30 min before primary antibody incubation at 4°C for 36–40 h. The sections were then washed with PBS and incubated for 3 h at room temperature with secondary antibodies diluted with the same buffer. Finally, the sections were washed in PBS, incubated with DAPI (1:10; Vector Laboratories, USA), mounted on gelatin-coated slides and covered with glycerol. Images were taken using a confocal microscope (Zeiss 800). The primary and secondary antibodies used were goat anti-BChE (1:500; R&D Systems, USA), rabbit anti-GFAP (1:500; Abcam, USA), rabbit anti-NeuN (1:400; Cell Signaling Technology, USA), Alexa Fluor 488-conjugated donkey anti-goat IgG (1:500; Life Technologies, USA), and Alexa Fluor 594-conjugated donkey anti-rabbit IgG (1:500; Life Technologies, USA). These antibodies were diluted with 0.1 M PBS, pH 7.4, containing 0.5% (w/v) BSA and 0.3% (v/v) Triton X-100 in PBS-T.

### Immunocytochemistry

C8D1A cells seeded in glass bottom dishes (Nunc™, USA) were fixed in 4% PFA for 15 min, permeabilized with 0.5% Triton X-100 in PBS for 20 min and blocked with 5% normal goat serum in PBS at room temperature for 30 min. The cells were subsequently incubated overnight in primary antibody diluted in 2% BSA at 4°C, incubated in secondary antibody for 2 h at room temperature, and stained with DAPI (Vector Laboratories, USA) for 5 min at room temperature. Immunofluorescence images were taken using a confocal microscope (LSM 800, Carl Zeiss, USA). The antibodies used were goat anti-BChE (1:50; R&D Systems, USA), rabbit anti-glial GFAP (1:100; Cell Signaling Technology, USA), Alexa Fluor 488-conjugated donkey anti-rabbit IgG (1:1,000; Life Technologies, USA), and Alexa Fluor 594-conjugated donkey anti-goat IgG (1:1,000; Life Technologies, USA).

### Golgi-Cox Impregnation

Mice were transcardially perfused with chilled 0.9% saline solution, and the brains were immediately removed. Following fixation, whole-brain Golgi-Cox impregnation was performed using a kit (GMS80020.2.vA, Genmed Scientifics Inc., USA) according to the manufacturer’s protocol, and serial brain sections (80-μm thick) were cut using a vibratome (VT 1000S, Leica). Using a microscope with a 100× oil objective (BX51, Olympus, Japan), dendritic spines on visually unobstructed apical and basal dendrites in the hippocampal CA1 region were imaged in a blinded manner. To capture the morphological extent of all spines on each dendritic segment of interest, a z-stack image series was acquired from the anterior to posterior limits of each spine set (incremental z-step distance, 0.25–0.5 μm). Spines with a neck were classified as either thin or mushroom-like, and those without a significant neck were classified as stubby. Spines with a neck were labeled thin or mushroom-like based on the head diameter (31). Quantitative morphological analysis of individual dendritic spines was performed using ImageJ (NIH, USA), and the spines were classified as thin or mushroom-like using previously described criteria (32). Spine analysis was performed by an experimenter blinded to the experimental conditions.

### Western Blotting

The hippocampal CA1 region was harvested after sodium pentobarbital (50 mg/kg, i.p.) anesthesia and decapitation. The tissues were then homogenized in RIPA buffer with freshly added protease inhibitors and centrifuged at 25,000 rpm for 25 min. Total protein was extracted from the C8D1A cells using cell lysis buffer containing protease & phosphatase inhibitors (Thermo Fisher Scientific, USA). Total protein concentrations were measured with a BCA assay kit (Fudebio-tech, China). Forty micrograms of total protein from each sample was denatured at 100°C for 10 min and separated by 8% SDS-PAGE. The proteins were then transferred to PVDF membranes (Millipore, USA), blocked in 5% skim milk and incubated overnight at 4°C with the following primary antibodies: goat anti-BChE (1:500; R&D systems, USA), rabbit anti-glutaminase (GLS) (1:1,000; Proteintech, USA), rabbit anti-glutamine synthetase (GS) (1:1,000; Proteintech, USA), rabbit anti-GAPDH (1:2,000; Cell Signaling Technology, USA), and rabbit anti-tubulin (1:1,000; Cell Signaling Technology, USA). Following washing, the blots were incubated with a horseradish peroxidase-conjugated secondary antibody (1:5,000; Cell Signaling Technology, USA) for 2 h at 37°C. The blots were developed with an enhanced chemiluminescence reagent (Millipore, USA) and digitally scanned using the ImageQuant Las 4000 Mini imaging system (GE, USA). The optical densities of the proteins were measured and normalized to the optical density (OD) of actin using ImageJ software (NIH, USA) in a blinded manner.

### ELISA for ACh Levels

ACh levels were measured using a human ACh ELISA kit (E4453-100, BioVision, Inc., USA) according to the manufacturer’s protocol. The tissue samples were handled at 4°C and homogenized after the addition of PBS, pH 7.4. The supernatants were collected after centrifugation for 20 min at 3,000 rpm. A total of 40-μl sample dilution buffer and 10-μl sample were added to each sample well. After mixing by gentle shaking, 100-μl HRP conjugate reagent was added to each well except the blank wells. Afterward, the plate was incubated for 60 min 37° after being sealed with a membrane and then rested for 30 s after the wash solution was discard. The washing procedure was repeated 5 times. Then, 50-μl Chromogen Solution A and 50-μl Chromogen Solution B were added to each well, and the plate was mixed by gentle shaking and incubated at 37°C for 15 min in the dark. Fifty microliters of stop solution was added to each well to terminate the reaction. The absorbance was read at 450 nm within 15 min after addition of the stop solution. The OD of the blank control well was set as zero.

### LV-BChE-RNAi Lentivirus Transfection

Mouse cloned astrocyte C8D1A cells were transfected with a negative control vector or LV-BChE-RNAi lentivirus (Shanghai Genechem Co., Ltd., China) according to the manufacturer’s protocol. A total of 2 × 10^5^ C8D1A cells were seeded in high-glucose DMEM containing 10% fetal bovine serum in 6-well culture plates (Corning Incorporated, USA) and incubated at 37°C for 24 h. The cells were then transfected by replacing the original culture medium with transfection medium containing the negative control vector (hU6-MCS-Ubiquitin-EGFP-IRES-puromycin) or LV-BChE-RNAi at a multiplicity of infection (MOI) of 100 for 16 h. The transfection medium was replaced with high-glucose DMEM containing 10% fetal bovine serum, and the cells were cultured for another 56 h.

### Glu, GS, and GLS Assays

Glu was extracted from brain tissues and cells by using a GLS assay kit (BC1580, Solarbio, China) according to the manufacturer’s protocol. Glu activity was calculated and measured by reading the absorbance at a wavelength of 340 nm with a microplate reader. Crude GS enzyme was extracted from brain tissues and cells using a GS assay kit (BC9015, Solarbio, China) according to the manufacturer’s protocol. The absorbance of the samples was read at 540 nm, and the activity of GS was calculated according to the absorbance value. A GLS assay kit (BC1450, Solarbio, China) was used to extract and quantify GLS according to the manual. A series of Gln standards (1, 0.8, 0.4, 0.2, 0.1, 0.05, 0.025, and 0 μmol/ml) was prepared from 10 μmol/ml GLS standard solution. The absorbances of both the samples and standard solutions were measured at 630 nm. A Gln standard curve with a linear equation (R^2^ > 0.99) was plotted. One unit of GLS is equivalent to the production of 1 μmol Gln in 1 gram of tissue at 37°C in 1 h. The activity of GLS was calculated based on the equation provided and is presented in units (U)/grams.

### Glutamine/Glutamate-Glo™ Assay

Gln and Glu levels in C8D1A cells were determined by the Glutamine/Glutamate-Glo™ Assay (J8022, Promega, USA). C8D1A cells were plated at a density of 10^5^ cells/well in high-glucose DMEM containing 10% fetal bovine serum in 6-well plates. After being transfected with LV-BChE-RNAi, both the transfected cells and medium were collected and processed following the protocol. A standard curve with a linear equation (R^2^ > 0.99) was plotted. A control group spiked with Gln or Glu was used to determine the recovery efficiency of Gln and Glu, respectively, and to calculate the Gln and Glu concentrations in the samples.

### Quantification and Statistical Analysis

The data are presented as the mean ± standard deviation unless otherwise indicated in the figure legends. The sample number (n) indicates the number of cells or mice used for each experiment and is specified in the figure legends. When the data met the assumptions of parametric statistical tests, the results were analyzed by Student’s t test. The data were tested for normality by using the Shapiro-Wilk test of normality and for homogeneity of variances by using Levene’s test for homogeneity of variances. All the statistical details of the experiments can be found in the results section. Differences in means were considered statistically significant at *p* < 0.05. Analyses were performed using IBM SPSS Statistics software (version 24).

## Results

### BChE Downregulation in the Hippocampal CA1 Region *In Vivo*

**Figure 1:**
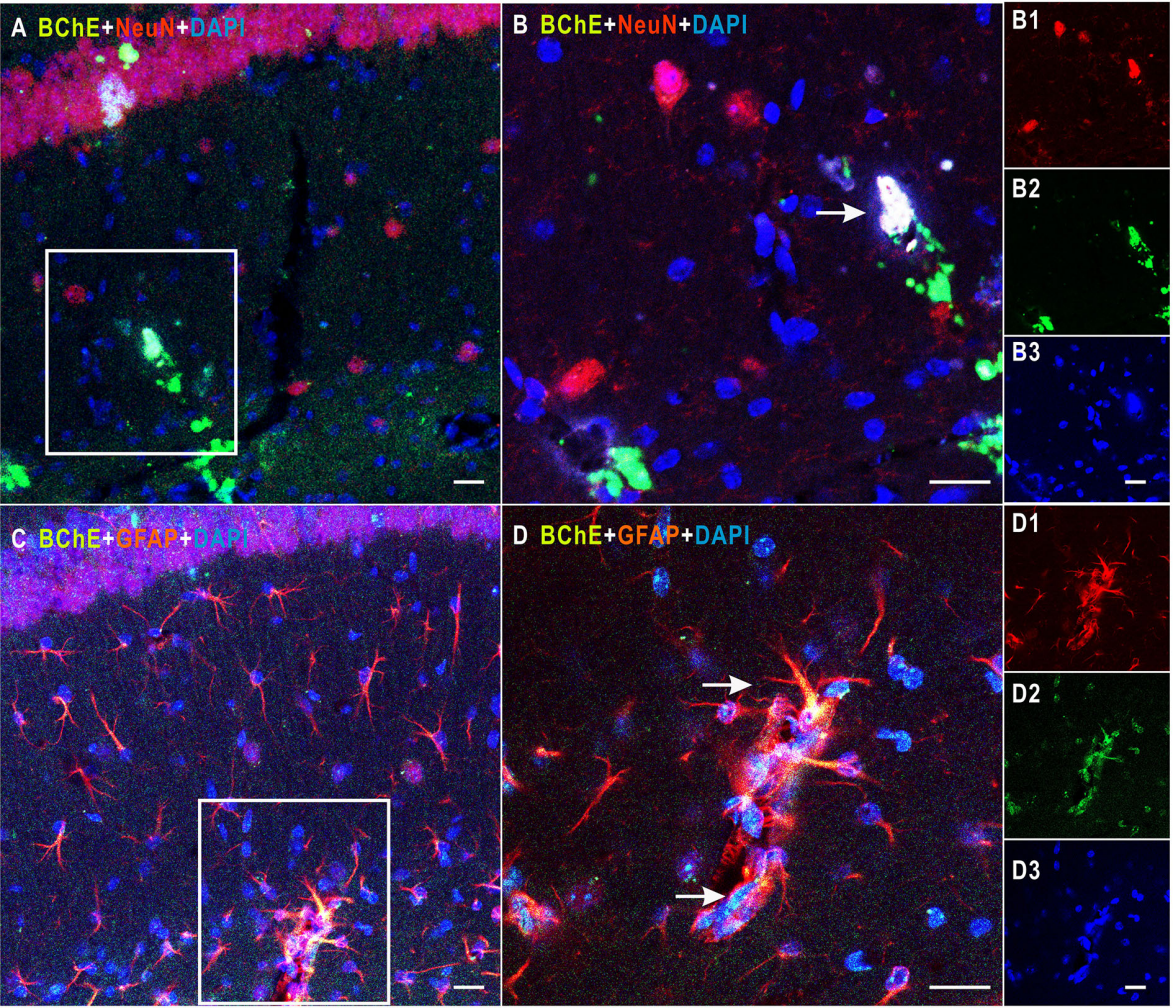
Detection of colocalization of BChE and GFAP or NeuN in the hippocampal CA1 area. **(A)** BChE (green), NeuN (red), and BChE-NeuN colocalization (yellow) were observed in the hippocampal CA1 region. **(B)** Picture **(B)** is a magnified image of the white box in **(A)**. B1–B3 shows single staining in **(B)**. BChE (green), NeuN (red), and DAPI (blue) staining are shown. **(C)** BChE (green), GFAP (red), and BChE-GFAP colocalization (yellow) were observed in neurons in the hippocampal CA1 region. **(D)** Picture D is a magnified image of the white box in **(A)**. D1–D3 shows single staining in **(B)**. BChE (green), GFAP (red), and DAPI (blue) are shown. Pictures **(A, C)**, **(B, D)**, and B1–B3 and D1–D3 are the same magnification. Scale bar: 20 μm.

**Figure 2:**
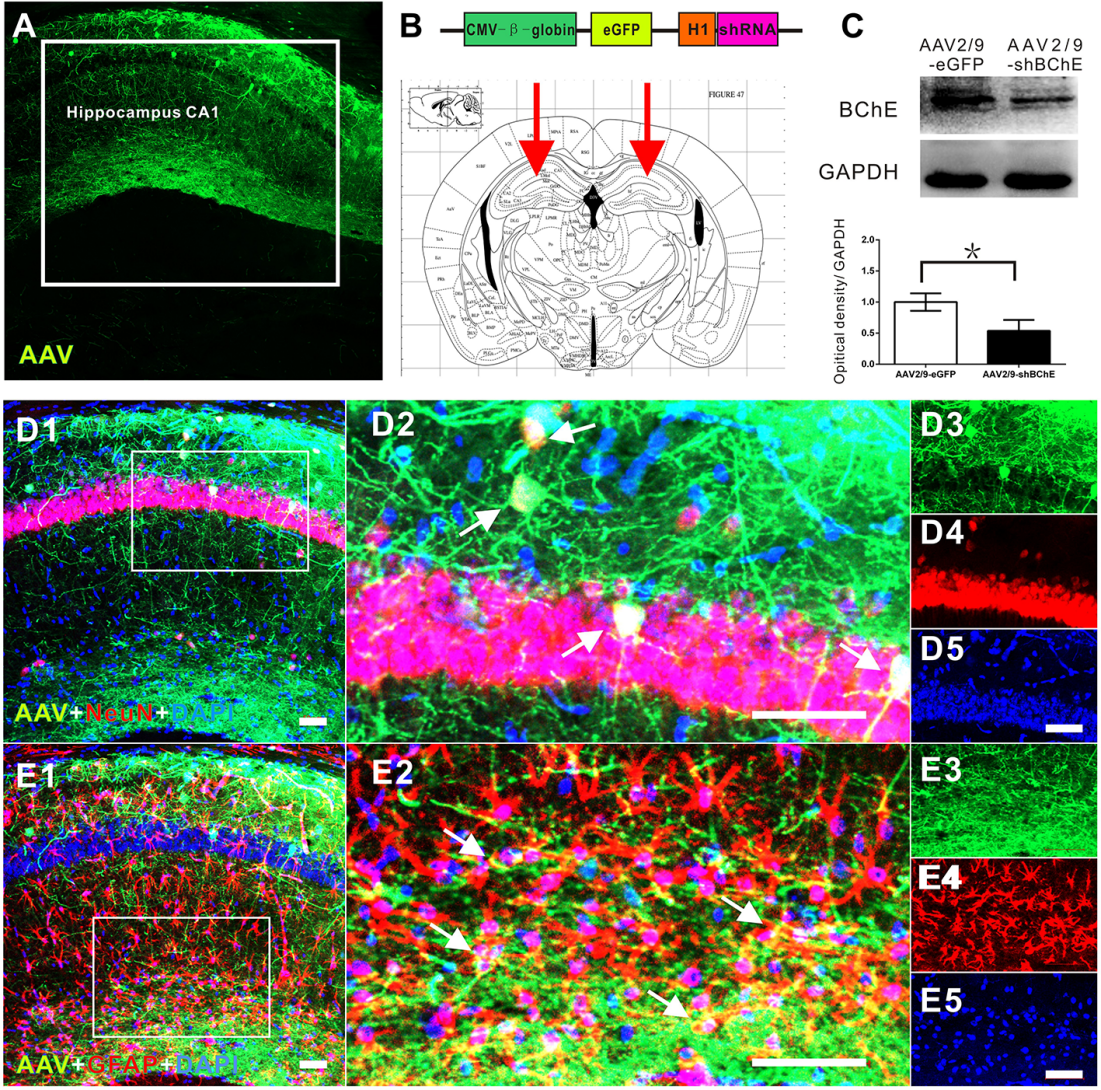
*In vivo* validation of decreased BChE expression induced by the BChE AAV vector. **(A)** Representative brain microsections showing AAV2/9-shBChE injection sites with and eGFP expression in the hippocampus CA1 region 3 weeks after injection. Scale bar: 100 µm. **(B)** Schematic of the shRNA-encoding AAV vector (top) and the injection site in the hippocampus CA1 region (bottom, representative mouse brain section, 2.0 mm AP from bregma). **(B)** AAV2/9-shBChE CMV, CMV immediate early promoter; β-globin, human β-globin intron; H1, H1 promoter. **(C)** Western blots for BChE. Animals were injected with either the AAV2/9-shBChE or control AAV2/9-eGFP vector (0.5 μl, 3×10^11^ vg/ml) into the unilateral hippocampal CA1 area. BChE shRNA induced robust deletion of endogenous BChE (*p < 0.05). **(D)** D1 is a representative brain microsections showing colocalization of BChE shRNA (eGFP) and NeuN (yellow) in the hippocampal CA1 region. D2 is a magnified image of the white box in D1. Single staining in D2 is shown on the right. AAV2/9-shBChE (green), NeuN (red) and DAPI (blue) are shown. **(E)** E1 are representative brain microsections showing colocalization of BChE shRNA (eGFP) and GFAP (yellow) in the hippocampal CA1 region. E2 is a magnified image of the white box in E1. Single staining in E2 is shown on the right. AAV2/9-shBChE (green), GFAP (red), and DAPI (blue) are shown. Scale bar: 50 µm. The data are presented as the mean ± SD.

In the present study, mice were divided into two groups: the AAV2/9-shBChE (BChE knockdown) group and the control AAV2/9-eGFP (control) group. BChE expression in whole mouse brain sections was examined by double immunofluorescence staining for BChE-GFAP and BChE-NeuN. BChE was distributed in the hippocampus and coexpressed with GFAP and NeuN (**Figure 1**), indicating that BChE is expressed in hippocampal CA1 astrocytes and neurons. We then unilaterally injected AAV2/9-shBChE to downregulate BChE expression and further evaluated the efficacy of the virus *in vivo*. The animals were injected with either the AAV2/9-shBChE or control AAV2/9-EGFP vector into the unilateral hippocampal CA1 region. Three weeks after AAV injection, brain sections were analyzed to measure cellular integrity in the hippocampus. For all animals, we found DAPI staining and strong eGFP expression, confirming robust transgene expression without toxicity due to surgery or viral transfection. AAV2/9-shBChE was distributed in the hippocampal CA1 region (**Figures 2A, B**), infecting both neurons and astrocytes (**Figures 2D–E**), and compared to the contralateral site which injected with AAV2/9-eGFP, it induced a statistically significant knockdown of endogenous BChE, as shown by western blot analysis (**Figure 2C**). There were no significant changes in BChE levels in the hippocampal CA1 region between the AAV2/9-eGFP injected side and the contralateral uninjected side, indicating that surgery and viral transfection did not affect BChE expression (**Supplementary Figure C**).

### BChE Knockdown in the Hippocampal CA1 Region Strengthened Contextual Fear Memory

**Figure 3:**
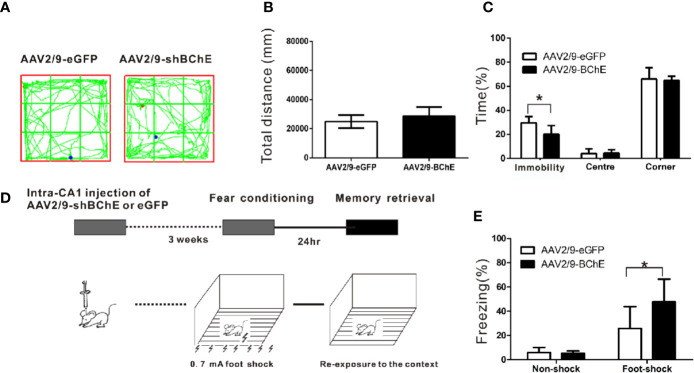
BChE knockdown in the hippocampal CA1 region strengthened contextual fear memory expression. **(A)** The locomotor trajectories of the AAV2/9-shBChE and control AAV2/9-eGFP groups in the open field. Representative exploratory traces are shown. **(B)** The total distance traveled in the open-field was similar between the AAV2/9-shBChE and control AAV2/9-eGFP groups when mice were tested for 24 h (*p* = 0.063; n = 6–7 mice per group). **(C)** The results showed a difference in immobility time between the two groups (*p* = 0.020, *p < 0.05; n = 6–7 mice per group). No significant difference in time spent in the center or time spent in the corner was observed between the AAV2/9-shBChE and control AAV2/9-eGFP groups (time spent in the center: *p* = 0.852; time spent in the corner: *p* = 0.769; n = 6–7 per groups). The data are presented as the mean ± SD. **(D)** Schematic of the procedure used in this experiment. **(E)** Percentage freezing increased due to BChE knockdown (*p* = 0.005, *p < 0.05; n = 13–14 mice per group). The results showed no significant difference between the two no shock groups (*p* = 0.704; n = 7 per group). The data are presented as the mean ± SD.

The behavior test was conducted 3 weeks after bilateral AAV injection in hippocampus CA1. The open-field test was performed to assess basal locomotor activity and evaluate the effect of AAV-mediated BChE knockdown in the hippocampal CA1 region. **Figure 3A** shows that there was no difference in the time spent in the center or the time spent in the corner between the mice transfected with AAV2/9-shBChE and those transfected with control AAV2/9-eGFP (time in the center: AAV2/9-eGFP, 4.11 ± 3.92% vs. AAV2/9-shBChE, 4.47 ± 2.83%, *t* test, *t* = 0.192, *p =*0.852; time in the corner: AAV2/9-eGFP, 66.10 ± 9.29% vs. AAV2/9-shBChE, 64.86 ± 3.56%, *t* test, *t* = −0.307, *p* = 0.769; n = 6–7 per group). **Figure 3B** also shows that there was no significant difference in the total distance traveled between the control AAV2/9-eGFP group (24896.23 ± 4406.7 cm) and AAV2/9-shBChE group (28730.39 ± 6101.52 cm) (*Mann-Whitney test*, *p* = 0.063; n = 6–7 per group). However, the AAV2/9-shBChE group exhibited a shorter total local immobility period (20.12 ± 7.24%) than the control group (29.53 ± 5.24%) (**Figure 3C**; *t* test, *t* = −2.714, *p* = 0.020; n = 6–7 per group) in the open-field test.

To determine whether BChE is involved in contextual fear memory, control AAV2/9-eGFP or AAV2/9-shBChE was bilaterally administered into the hippocampal CA1 region of the mice 3 weeks before fear conditioning. Twenty-four hours after the conditioning sessions, the mice were re-exposed to the context to elicit fear memory (**Figure 3D**). Re-exposure to the context elicited a fear response known as freezing, which serves as a fear memory indicator. There was no difference in the duration of the freezing period between the two “no-shock” control groups, namely, the AAV2/9-eGFP group (5.87 ± 4.29%) and the AAV2/9-shBChE group (5.17 ± 2.08%) (**Figure 3E**; *t* test, *t* = 0.390, *p* = 0.704; n = 7 per group). This validates that the foot-shock groups exhibited a fear response even though the AAV2/9-shBChE group showed a shorter immobility period in the open-field test. **Figure 3E** illustrates that compared to the control group (25.81 ± 17.90%), the group in which BChE was knocked down in the hippocampal CA1 region by AAV exhibited a longer freezing period (47.68 ± 18.85%), which indicates that lower BChE expression can strengthen fear memory retention (*t* test, *t = −*3.091, *p* = 0.005; n = 13–14 per group).

### BChE Knockdown in the Hippocampal CA1 Region Significantly Increased the Total Spine Density of Pyramidal Dendrites After CFC

**Figure 4:**
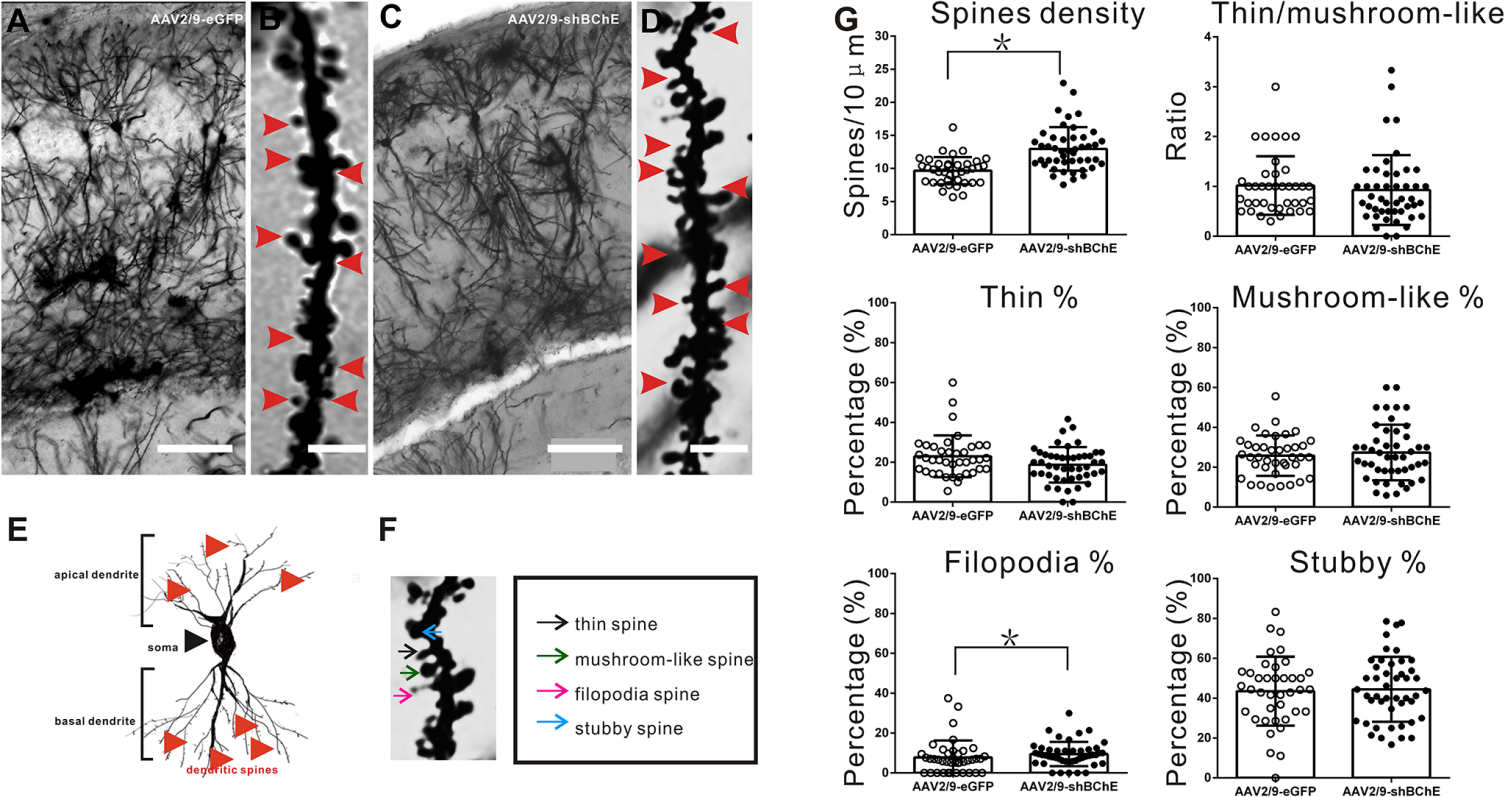
BChE knockdown in the hippocampal CA1 region significantly increased the total spine density on pyramidal dendrites after CFC. **(A**–**D)** Representative Golgi-Cox impregnated spines in the hippocampal CA1 area in the AAV2/9-shBChE and control AAV2/9-eGFP groups 24 h after training. A and C, scale bar: 100 μm. B and D, scale bar: 2.5 μm. **(E)** Representative images showing the morphology of apical and basal spines. **(F)** Representative images of the morphology of thin, mushroom-like, filopodia and stubby spines. **(G)** Quantification of the total density of spines in the AAV2/9-shBChE and control AAV2/9-eGFP groups. Significant differences were observed between the groups (*p <* 0.001, *p < 0.05; n = 39–46 dendrites from 3 mice per group). The results also showed that the percentage of filopodia spines increased when BChE was knocked down (*p* = 0.038, *p < 0.05; n = 39–46 dendrites from three mice per group). The data are presented as the mean ± SD.

To further examine whether BChE knockdown elicits synaptic plasticity changes during fear memory formation, Golgi-Cox impregnation was used to investigate the morphology of dendritic spines on hippocampal CA1 pyramidal neurons. The hippocampus was removed and prepared for Golgi-Cox impregnation after contextual fear memory assessment. AAV-mediated BChE knockdown in the hippocampal CA1 region increased the total spine density of hippocampal CA1 pyramidal basal dendrites, as illustrated in **Figures 4A–D**, G (AAV2/9-eGFP: 9.67 ± 2.06/10 µm, AAV2/9-shBChE: 12.96 ± 3.30/10 µm, *p <* 0.001, *t* test; n = 39–46 dendrites from three mice per group). Representative images of a single dendrite and the spine morphology of thin, mushroom-like, filopodia, and stubby spines, as observed by Golgi-Cox staining, are shown in **Figures 4E, F**. These four types of dendritic spines were quantified, and there were significantly more filopodia spines on hippocampal neurons in the BChE knockdown group than in the control group, as shown in **Figure 4G** (AAV2/9-eGFP: 7.76 ± 8.49%, AAV2/9-shBChE: 9.50 ± 6.09%, *p* = 0.038, *Mann-Whitney* test; n = 39–46 dendrites from three mice per group). There were no significant differences in the percentage of thin spines, mushroom-like spines, or stubby spines between the AAV2/9-eGFP and AAV2/9-shBChE groups (**Figure 4G**; thin spines, AAV2/9-eGFP: 22.94 ± 10.49%, AAV2/9-shBChE: 18.73 ± 8.90%, *p* = 0.092, *Mann-Whitney* test; mushroom-like spines, AAV2/9-eGFP: 25.00 ± 9.00%, AAV2/9-shBChE: 27.35 ± 13.97%, *p* = 0.354, *t* test; stubby spines, AAV2/9-eGFP: 43.48 ± 17.28%, AAV2/9-shBChE: 44.43 ± 16.28%, *p* = 0.989, *Mann-Whitney* test). Additionally, the ratio of thin to mushroom-like spines was not different between the two groups (**Figure 4G**; AAV2/9-eGFP: 1.02 ± 58.80, AAV2/9-shBChE: 0.9 3± 0.70, *p* = 0.297, *Mann-Whitney* test).

### BChE Knockdown in the Hippocampal CA1 Region Significantly Increased Glu and GS Enzyme Activity in the Hippocampal CA1 Region After CFC

**Figure 5:**
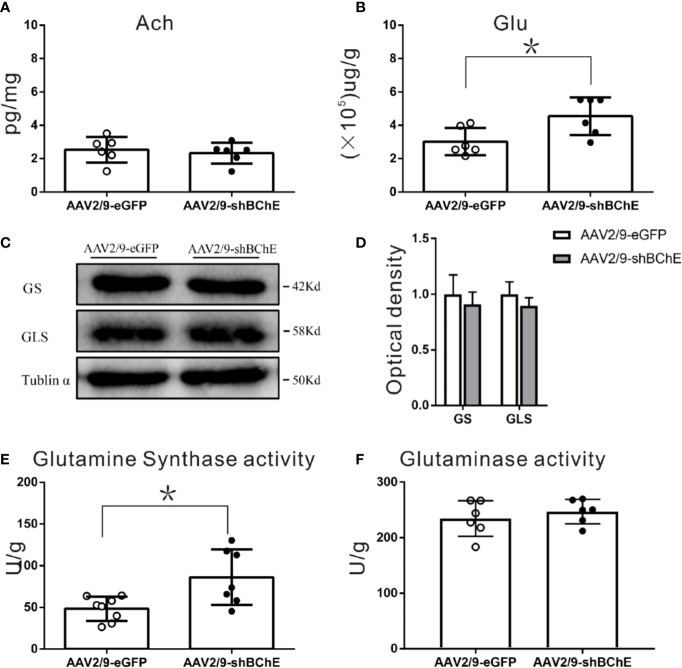
BChE knockdown in the hippocampal CA1 region significantly increased Glu and GS enzyme activity in the hippocampal CA1 region after CFC. **(A)** Quantification of the concentration of ACh in the AAV2/9-shBChE and AAV2/9-eGFP groups. No significant differences were observed between the groups (p = 0.622; n = 6 mice per group). **(B)** The results showed a significant difference in Glu concentration between the two groups (*p* = 0.015, *p < 0.05; n = 6 mice per group). **(C, D)** Western blots for GS and GLS. Three weeks following surgery, mice were sacrificed, and hippocampal CA1 tissues were analyzed by western blotting. There were no significant differences between the two groups. **(E, F)** Quantification of GS and GLS activities in the AAV2/9-shBChE and AAV2/9-eGFP groups. The results showed that only the enzyme activity of GS significantly was increased in the BChE knockdown group compared with the negative control group (*p* = 0.024, *p < 0.05; n = 7–8 mice per group). The data are presented as the mean ± SD.

Both ACh and Glu levels were evaluated to elucidate the potential mechanism that caused the higher dendritic spine density and longer fear memory retention observed when BChE was knocked down by AAV in the hippocampal CA1 region. Hippocampal CA1 tissues were harvested after behavioral tests, and there was no difference in ACh levels between the control AAV2/9-eGFP (2.54 ± 0.78 pg/mg) and AAV2/9-shBChE groups (2.33 ± 0.63 pg/mg) (**Figure 5A**; *p* = 0.622, *t* test; n = 6). In contrast, compared to normal BChE expression in the AAV2/9-eGFP group (2.80 ± 0.69 (×10^5^) µg/g), BChE knockdown in the hippocampal CA1 region significantly increased the Glu concentration [4.55 ± 1.13 (×10^5^) µg/g] (**Figure 5B**; *p* = 0.015, *t* test; n = 6).

To further identify the factors in the Glu-Gln cycle that contribute to fear memory formation, we examined alterations in the expression and enzyme activity of key enzymes, namely, GS and GLS, in the hippocampal CA1 region. GS and GLS are the key enzymes in the Glu-Gln cycle. Western blot analysis of both groups (**Figures 5C, D**) showed no aberrant protein expression of GS (AAV2/9-eGFP: 1.00 ± 0.17 vs. AAV2/9-shBChE: 0.91 ± 0.11, *p = 0.491*, *t* test; n = 3) or GLS (AAV2/9-eGFP: 1.00 ± 0.11, AAV2/9-shBChE: 0.90 ± 0.07, *p = 0.251*, *t* test; n = 3). However, GS enzyme activity was higher in the AAV2/9-shBChE group (86.31 ± 33.29 U/g) than in the control AAV2/9-eGFP group (48.39 ± 14.41 U/g) (**Figures 5E, F**; *p* = 0.024, *t* test; n = 7–8), and no significant difference in GLS enzyme activity was observed between these two groups (AAV2/9-eGFP: 234.57 ± 32.10 U/g, AAV2/9-shBChE: 246.98 ± 22.13 U/g, *p = 0.454*, *t* test; n = 6).

### *In Vitro* BChE Knockdown in Astrocytes Increased Both Glu and Gln Levels and GS Enzyme Activity

**Figure 6:**
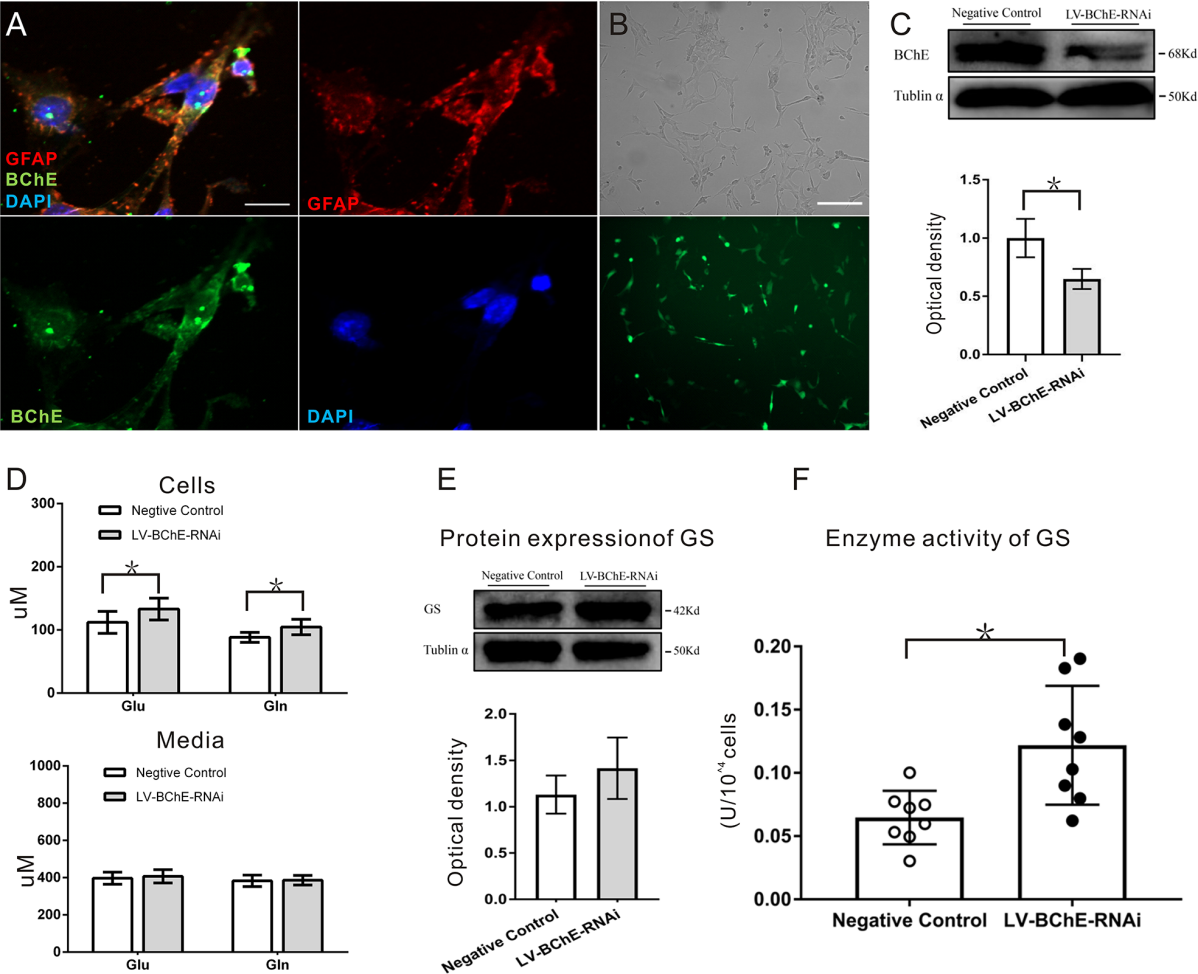
*In vitro* BChE knockdown in astrocytes increased both Glu and Gln levels and GS enzyme activity. **(A)** Colocalization of the astrocyte marker GFAP (red) and BChE (green) was detected in C8D1A cells, Scale bar: 20 µm). **(B)** Representative images of EGFP expression 72 h after C8D1A cells were transfected with lentivirus. Scale bar: 200 μm. **(C)** Western blots probed with anti-BChE and anti-tubulin α antibodies. The expression of BChE was signiﬁcantly decreased 72 h after lentivirus transfection in the LV-BChE-RNAi group compared with the negative control group (*p* = 0.031, *p < 0.05; n = 3). **(D)** The results showed that both Glu and Gln levels were increased when BChE expression was downregulated (Glu: *p* = 0.036; n = 6–8; Gln: *p* = 0.010; n = 8; *p < 0.05). However, the data showed that the concentrations in the medium were not significantly different between the negative control and LV-BChE-RNAi groups (Glu: *p* = 0.566; n = 8; Gln: *p* = 0.834; n = 8). **(E)** Representative western blot images of GS expression in C8D1A cells after BChE knockdown are shown. The protein expression of GS was not significantly changed after BChE knockdown in the LV-BChE-RNAi group compared with the negative control group (*p* = 0.275, n = 3). Relative intensity: GS/tubulin α. **(F)** BChE knockdown signiﬁcantly increased the enzyme activity of GS in C8D1A cells in the LV-BChE-RNAi group compared with the negative control group (*p* = 0.003, *p < 0.05; n = 8). The data are presented as the mean ± SD.

We utilized C8D1A cells, a mouse astrocyte cell line, to further characterize the effect of BChE knockdown on the Glu-Gln cycle. By using immunofluorescence staining, we confirmed that BChE was expressed in GFAP-positive C8D1A cells (**Figure 6A**). We then used a lentivirus (LV-BChE-RNAi) to knockdown BChE in C8D1A cells. The cells were divided into two groups: the LV-BChE-RNAi group and the negative control group. C8D1A cells in the negative control group were transfected with the hU6-MCS-Ubiquitin-EGFP-IRES-puromycin vector. Lentivirus expression was consistently observed in C8D1A cells 72-h posttransfection (**Figure 6B**), and western blot analysis showed a significant reduction in BChE protein expression in the LV-BChE-RNAi group (0.65 ± 0.05) compared to the negative control group (1.00 ± 0.10) (**Figure 6C**; independent-samples *t* test, *p* = 0.031; n = 3).

As astrocytes readily convert Glu to Gln *via* the GS pathway and release Gln extracellularly, we tested the concentrations of Glu and Gln in both cell lysates and the culture medium 72-h posttransfection, and the results are shown in **Figure 6D**. Higher Glu and Gln protein levels were found cell lysates from the LV-BChE-RNAi group compared to those from the negative control group (Glu conc.: negative control vs. LV-BChE-RNAi: 112.01 ± 17.40 µM vs. 133.08 ± 17.32 µM, p = 0.036; n = 6–8; Gln conc.: negative control vs. LV-BChE-RNAi: 88.27 ± 7.81 µM vs. 104.53 ± 12.26 µM, *t* test, p = 0.010; n = 8), while no differences were detected in the culture medium (Glu conc.: negative control vs. LV-BChE-RNAi: 397.01 ± 32.54 µM vs. 407.10 ± 35.99 µM, *p* = 0.566; n = 8; Gln conc.: negative control vs. LV-BChE-RNAi: 383.14 ± 26.07 µM vs. 386.17 ± 26.07 µM, *t* test, *p* = 0.834; n = 8). The increased concentration of Glu and Gln in BChE knockdown C8D1A cells compared to control cells was accompanied by significantly higher GS enzyme activity (negative control vs. LV-BChE-RNAi: 0.061 ± 0.008 vs. 0.122 ± 0.017, *t* test, *p* = 0.003; n = 8), but there were no expression changes (negative control vs. LV-BChE-RNAi: 1.131 ± 0.119 vs. 1.415 ± 0.191, *t* test, *p* = 0.275; n = 3) compared to the negative control (**Figures 6E, F**). GS expression and enzyme activity observed in BChE knockdown astrocytes is consistent with those *in vivo* when BChE was knocked down in the hippocampal CA1 region of mice.

## Discussion

The major finding of the present study is that lower BChE expression combined with contextual fear memory increases total spine density and the percentage of filopodia spines on pyramidal dendrites. These changes are accompanied by elevated Glu levels and GS enzyme activity, but not alterations in ACh levels, this where various clinical and preclinical studies have noted the involvement of ACh in the neurobiology and treatment of PTSD (8–10). *In vitro* BChE knockdown in an astrocyte cell line further verified the increases in intracellular Glu and Gln levels and GS enzyme activity. While this study is limited by the omission of a non-shocked group in the experiment where the findings could be a combination of the fear conditioning protocol and BChE knockdown, the neurobiological changes observed are likely due to the lower BChE expression in the knockdown group. Although we need to further confirm the contribution of lower BChE expression on the observed neurobiological changes, this study provides new insight into the regulatory role of BChE on cognition and suggests a potential relationship between BChE and PTSD pathophysiology.

### BChE Knockdown in the Hippocampal CA1 Region Strengthens Contextual Fear Memory Expression and Increases the Dendritic Spine Density and Percentage of Filopodia Spines

The pathophysiology of PTSD remains unclear. We explored the function of BChE in fear memory to help elucidate the pathological mechanism of PTSD. In the present study, a long-acting adeno-associated virus with low toxicity that has been widely used in the hippocampal CA1 region to downregulate BChE expression was injected into mice. We confirmed the efficacy of this virus based on low BChE expression. Fear conditioning in AAV2/9-shBChE mice can mimic PTSD flashbacks to a certain extent when BChE expression in the hippocampal CA1 region is low.

Our data from the contextual fear memory experiments showed a significant increase in freezing percentage when BChE was downregulated in the hippocampal CA1 region. This increase was not attributable to locomotor activity or general activity changes because no significant difference was found in open-field test performance. Indeed, although there is no statistical difference in the locomotor activity between the AAV2/9-eGFP and AAV2/9-shBChE group, the average distance is higher in AAV2/9-shBChE group. This phenomenon may be resulted from the decreased fear memory induced-immobility, and the mice were also more active in the open field. This inconsistency between the open-field and the fear memory induced-immobility is not the only case observed so far. Another study also showed the same phenomenon in the commensal bacteria-bearing specific pathogen free mice and germ free mice. It highlighted the importance of microbiota in normal brain morphological development and maturation that involved behavioral outcomes such as cognitive function and locomotion. In the current study, the neurobiological changes reported might be attributed by the effect of BChE knockdown in hippocampal CA1 region; however, this will require further investigation (33).The observed strengthening of contextual fear memory is consistent with reports on mouse models of manganese-induced neurotoxicity, mouse models of stress, AD patients and animal models of AD; low BChE expression and decreased BChE function are associated with higher cognitive ability and higher stress levels (2, 4–7, 34). On the other hand, a cognitive study in BChE transgenic mice demonstrated that BChE knock-out mice show better memory performance, and suggested that the hippocampus CA1 BChE might not only has function on fear memory, but also in other memory types (1). The results further showed that the dendritic spine density, especially the percentage of filopodia spines, was increased in the hippocampal CA1 region. These results are in agreement with the improvements in retention by BChE−/− AD model mice (1). Studies have shown that low BChE expression is accompanied with a series of high stress-related behaviors which has been known to impair cognition (5, 6). Although the relationship between BChE and glucocorticoids is still unclear, BChE has been known to be associated with one of the stress hormone, ghrelin. BChE has displayed a potential physiological role in ghrelin inactivation (6, 7). High stress level has shown to deteriorate the fear memory and lead to fear memory generalization (35). A study proposed that the foot-shock should be conducted under 0.8 mA, or else it would be easier to induce fear memory generalization from the glucocorticoids effect (35). Therefore, a foot-shock intensity of 0.7 mA is used in this study in order to minimize the contribution of the stress hormone. Long-term memory (LTM) formation is a process accompanied by structural changes at synapses and increased spine density (36). Filopodia spines present a significant advantage in establishing functional synaptic connections. An increased percentage of filopodia spines can lower the threshold and reduce the time to form new dendritic spines and synapses, providing a substrate for fast learning (37). These morphological changes in spines provide direct evidence that contextual fear memory is strengthened when BChE is downregulated.

### BChE in the Hippocampal CA1 Region Might Modulate Fear Memory and Increase Glu Levels

BChE is known to have a vicarious effect on ACh hydrolysis in neurodegenerative diseases. We found that BChE knockdown in the hippocampal CA1 region exerts no effect on ACh levels. This indicates that BChE expression affects memory formation in an ACh-independent manner when AChE concentrations are normal. This finding is consistent with previous findings showing that a BChE-selective inhibitor elevates ACh levels in AChE knockout mice but has no effect on wild-type mice (29). A study showed that the axonal filopodia spines in cultured neurons show rapid outgrowth and higher dendritic spine density when induced by Glu or ACh (38). In addition, Glu can also trigger morphological changes in immature protrusions in hippocampal slice cultures (39). Spines are associated with most Glu excitatory synaptic inputs, and their growth is modulated by Glu in the brain (39). We detected an increase in the neurotransmitter Glu in the hippocampus CA1 after fear memory retrieval. Glu is known as a key excitatory neurotransmitter in the processes of learning and memory, is released from presynaptic nerve terminals and interacts with postsynaptic receptors, such as N-methyl-D aspartate (NMDA) receptors (26). Clinical studies of PTSD have also shown the involvement of Glu in the pathological process (18, 19). Bordi et al. demonstrated that metabotropic glutamate receptor and NMDA receptor have different impact on learning and memory, specifically the metabotropic glutamate receptor which may require for only a restricted subset of spatial learning tasks. In the same study, Bordi et al. found that NMDA receptor may play an integral role in all spatial learning such as contextual fear memory (40). These shows that high Glu level that caused by BChE knockdown might contribute to the strengthened contextual fear memory expression *via* NMDA receptor. The present findings are aligned with previous studies showing that an elevated Glu level is accompanied by a higher filopodia spine density and enhanced memory performance (20, 37).

Most Glu in the brain is produced by the Glu-Gln cycle, which has been verified in fear memory formation studies (23, 41, 42). Both GS and GLS are key enzymes in the Glu-Gln cycle. They play important roles in the pathological mechanisms of a variety of neuropsychiatric disorders (25, 43). A reduction in BChE expression elevates GS enzyme activity without affecting its expression, which indicates that BChE has a regulatory effect on the Glu-Gln cycle. GS is exclusively expressed in astrocytes and modulates the Gln-Gln cycle by synthesizing Gln from Glu (44). Recent studies have revealed that insufficient GS activity during synaptogenesis causes significant spatial memory impairment in adult mice (44). Thus, we speculate that the downregulation of BChE increases the Glu-Gln cycle, which may rely on the function of GS. The lack of observable changes in neuronal GLS expression and enzyme activity in the present study suggests that BChE targets the GS pathway of the Glu-Gln cycle in astrocytes.

### BChE Affects GS Enzyme Activity in Astrocytes

The regulatory role of BChE in the GS pathway of the Glu-Gln cycle in astrocytes was assessed in the C8D1A astrocyte cell line. In the brain, Glu released from neurons is taken up from the synaptic cleft by astrocytes through Glu transporter-1 (GLT-1) and then converted to Gln by GS through the Glu-Gln cycle (44–47). While there were no changes in the culture medium, we observed higher intracellular Glu and Gln protein levels when BChE was expressed at low levels. The difference between levels in cell homogenates and culture medium indicates that BChE might be involved in the synthesis of Gln rather than the process of astrocytic uptake. We propose that the increased levels of Glu and Gln may be due to higher activity of the tricarboxylic acid cycle (TCA), which converts glucose to Glu, in astrocytes (24, 25). However, this may need further verification. Additionally, the increased GS enzyme activity observed in BChE knockdown astrocytes was consistent with the findings of our animal experiments. Hence, it is reasonable to believe that downregulation of BChE may also promote the Glu-Gln cycle due to improvements in GS function.

In conclusion, the present study supports a relationship between BChE in the hippocampal CA1 region and contextual fear memory, in which low BChE expression enhances contextual fear memory and promotes the Glu-Gln cycle in astrocytes. This may be the first time that BChE has been found to be involved in Glu-Gln cycle activity. Further investigation into how BChE affects the Glu-Gln cycle between neurons and astrocytes is warranted. This study highlights a potential perturbed mechanism in neurological disorders, such as PTSD, while contributing to the understanding of the function of BChE function in the human brain.

## Data Availability Statement

All datasets presented in this study are included in the article/supplementary material.

## Ethics Statement

The animal study was reviewed and approved by The Animal Care and Use Committee of Sun Yat-Sen University.

## Author Contributions

SC, K-LT, and WT are involved in conceptualization of the study. SC, ZL, WS, and QT performed the contextual fear memory and open-field tests. SC and RC were involved in the morphological test. K-LT and ZL were involved in western blot. ZL and K-LT were involved in *in vitro* experiments. SC and ZL were involved in analysis of the dataset. SC, K-LT, and HZ contributed to the study resources. SC, K-LT and WT contributed to the funding acquisition. The draft manuscript was written, reviewed, and edited by SC, ZL, and K-LT. All authors contributed to the article and approved the submitted version.

## Funding

This work was supported by the Science and Technology innovation Project of Foshan (Grant No. 2017IT100162), Postdoctoral Science Foundation of China (Grant No. 2017M622644), Natural Science Foundation of China (Grant No. 81850410549) and Guizhou Science and Technology Cooperation Platform Talents Project (Grant No. [2018]5772-022).

## Conflict of Interest

The authors declare that the research was conducted in the absence of any commercial or financial relationships that could be construed as a potential conflict of interest.
