# Supplementary material for: Enhanced Contextual Fear Memory and Elevated Astroglial Glutamate Synthase Activity in Hippocampal CA1 BChE shRNA Knockdown Mice
Source: Front Psychiatry. 2020 Sep 11;11:564843. doi: 10.3389/fpsyt.2020.564843 (PMC7518375; doi:10.3389/fpsyt.2020.564843)
Supplement: Supplementary file 1 [file DataSheet_1.pdf]

Supplementary Material

1 Supplementary Data

Strong eGFP distribution was observed in the hippocampal CA1 region after AAV2/9- eGFP injection into this area (Fig. A-B). Western blot analysis showed no significant changes in BChE levels between the injected hippocampal CA1 region and the contralateral uninjected region (Fig. C), indicating that surgery and viral transfection did not affect BChE expression.

2 Supplementary Figures and Tables

2.1 Supplementary Figures

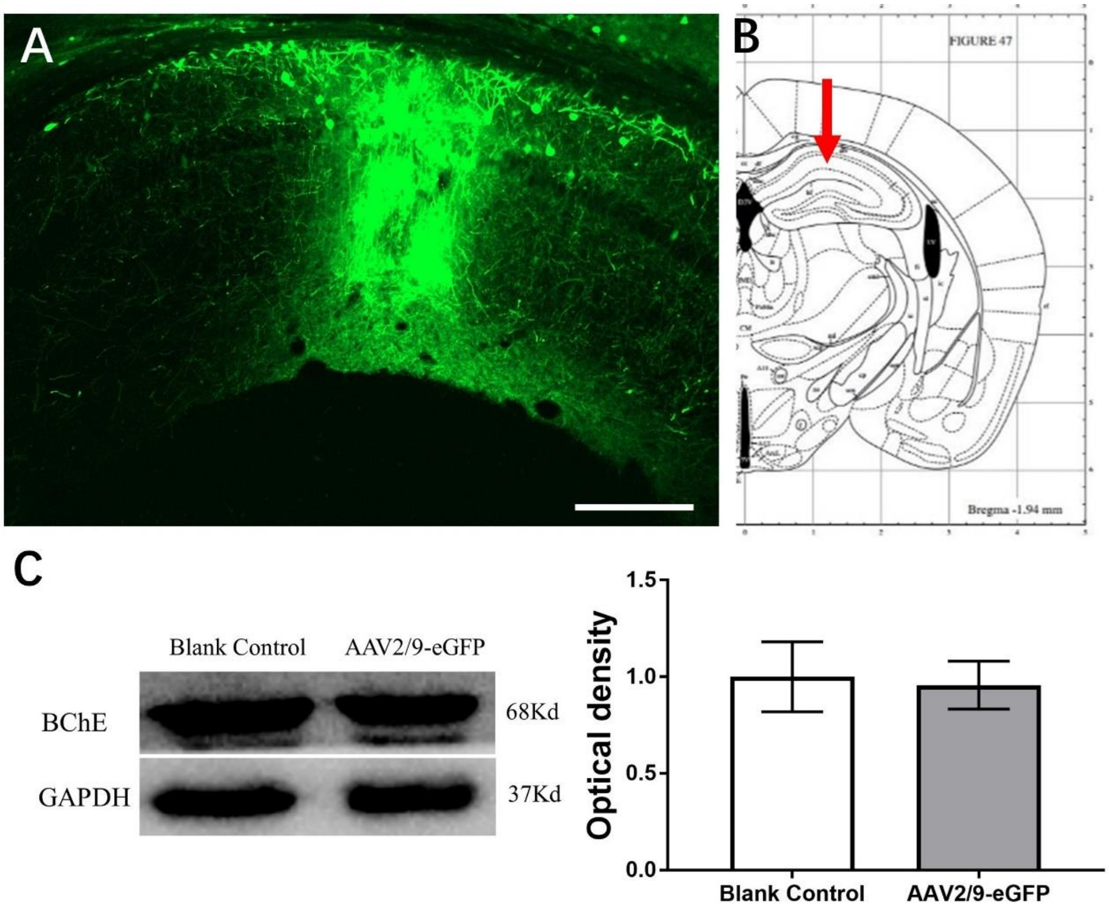

Supplementary Figure 1. (A) Representative image showing the AAV2/9-eGFP injection site and eGFP expression in the hippocampal CA1 region. Scale bar: 200 μm. **(B)** The injection site in the hippocampus CA1 region**.(C)** The protein expression of BChE, as evaluated by western blotting, was not significantly different between the two groups (blank control vs AAV2/9-eGFP: 1 ± 0.10 vs 0.96 ± 0.07, p=0.76; n=3)
